# Atlas Florae Europaeae notes, 35. Further critical notes on Cytisussect.Tubocytisus (Fabaceae) in Europe

**DOI:** 10.3897/phytokeys.238.118032

**Published:** 2024-02-23

**Authors:** Alexander N. Sennikov, Valery N. Tikhomirov

**Affiliations:** 1 Botanical Museum, Finnish Museum of Natural History, University of Helsinki, Helsinki 00014, Finland University of Helsinki Helsinki Finland; 2 Belarusian State University, Minsk, Belarus Belarusian State University Minsk Belarus

**Keywords:** Balkans, *Chamaecytisus*, Leguminosae, nomenclature, synonymy, taxonomy, typification

## Abstract

A few species names in Cytisussect.Tubocytisus are re-assessed and taxonomically evaluated. Diagnostic characters are discussed and the species status of *C.absinthioides* Janka, *C.eriocarpus* Boiss., *C.frivaldszkyanus* Degen, *C.jankae* Velen. and *C.smyrnaeus* Boiss. is confirmed. The holotype of *Cytisustriflorus* Lam. was found to belong to *C.hirsutus* L. rather than to the *C.ratisbonensis* group as currently treated. *Cytisuslasiosemius* Boiss. is not the correct name for *C.frivaldszkyanus* Degen, but another synonym of *C.hirsutus*. *Cytisuslitwinowii* V.I.Krecz., which was known solely from the holotype, is a synonym of *C.austriacus* L. s.str. *Chamaecytisuspseudojankae* Pifkó & Barina, reported from a small area shared between Albania, Greece and North Macedonia, is treated as a subalpine variant of *C.austriacus*. *Cytisustmoleus* Boiss. is removed from the synonymy of *C.eriocarpus* and added to the synonymy of *C.pygmaeus* Willd. Cytisusfalcatussubsp.albanicus Degen & Dörfl. and *C.pubescens* Gilib. are synonymised with *C.hirsutus*. *Cytisusmicrophyllus* Boiss. is moved from *C.austriacus* s.l. to the synonymy of *C.frivaldszkyanus*, and *C.pindicola* (Degen) Halácsy to the synonymy of *C.jankae*. *Chamaecytisuscalcareus* (Velen.) Kuzmanov is accepted as *Cytisuscalcareus* (Velen.) Sennikov & Val.N.Tikhom., **comb. nov.**, and its distribution is circumscribed. Cytisushirsutusvar.ciliatus (Wahlenb.) Hazsl. and C.polytrichusvar.subglabratus Val.N.Tikhom. & Sennikov, **var. nov.** are recognised as glabrous variants of the corresponding species. Lectotypes of *C.ciliatus*, *C.hirsutissimus* K.Koch, *C.jankae*, *C.lasiosemius*, *C.pubescens*, *C.rhodopeus* J.Wagner ex Bornm. and *C.thirkeanus* K.Koch are designated. *Cytisuspolytrichus* is reported from the Western Caucasus in place of *C.wulffii* auct.

## Introduction

The genus *Cytisus* Desf. nom. cons. is one of the largest genera of tribe Cytiseae Bercht. & J.Presl (Talavera and Salgueiro 1999). Its circumscription is still uncertain due to the lack of modern phylogenetic work; old phylogenies (Cubas et al. 2002; Pardo et al. 2004) indicated unresolved relationships in the *Cytisus*-group in Cytiseae, with some taxa being currently treated as segregate genera *Adenocarpos* DC. or *Argyrocytisus* (Maire) Raynaud, *Calicotome* Link, *Chamaecytisus* Link, *Cytisophyllum* O.Lang (e.g. Talavera and Salgueiro (1999); Freiberg et al. (2020); Govaerts et al. (2021)). Due to unresolved relationships with and a morphological similarity of these groups to the core lineages of *Cytisus*, a broad circumscription of *Cytisus* s.l. was advocated by taxonomic experts (Cristofolini 1991; Cristofolini and Conte 2002; Cristofolini and Troía 2006, 2017) and is followed here.

Cytisussect.Tubocytisus DC. (= *Chamaecytisus* Link) is the largest part of *Cytisus* s.l. Its species number varies greatly according to the accepted concept, ranging from about 30 (Cristofolini and Troía 2006) to 43 (Govaerts et al. 2021). The species in this group may be very similar to each other, being different in minor characters of dimensions and pubescence (Cristofolini 1991; Cristofolini and Troía 2017). This fact poses a natural difficulty in the taxonomic delimitation of this group and is responsible for wide discrepancies and contradictions in taxonomic assessments between individual researchers (e.g. Gibbs (1970); Tzvelev (1987); Cristofolini (1991); Pifkó (2009)).

Published treatments of Cytisussect.Tubocytisus varied in detail, but remained consistent in one major feature, i.e. a high level of taxonomic splitting, resulting in narrowly delimited taxa with faint, but constant differences in pubescence, dimensions, leaf shape and habit (Sennikov and Tikhomirov 2024a). Certain deviations observed between particular treatments may be better explained by some material being inaccessible to individual researchers, thus accounting for lumping of single species or misinterpretation of particular species names.

In the present contribution, we provide notes on some species of *Cytisus*, mostly in Central and Eastern Europe and the Balkans, which require taxonomic or nomenclatural corrections. This study is based on our examination of the original material and protologues of relevant species names, which allowed us to match otherwise discrepant taxonomic decisions made by various researchers (e.g. Gibbs (1970); Cristofolini (1991); Pifkó (2005, 2009); Pifkó and Barina (2016)).

The scope of this study is limited to a selection of species belonging to three groups of C.sect.Tubocytisus, i.e. *C.hirsutus* (stems erect or prostrate, inflorescences terminal and lateral, leaflets elliptic-lanceolate to oblanceolate, hairs long patent), *C.austriacus* (stems erect, inflorescences terminal, leaflets lanceolate, apically narrowed, hairs strigose, mostly appressed), *C.pygmaeus* (stems ascending, inflorescences terminal, leaflets elliptic-lanceolate to obovate-lanceolate, hairs long and short, appressed, subpatent or patent). One more species-rich and taxonomically problematic group, *C.ratisbonensis*, is treated separately elsewhere (Sennikov and Tikhomirov 2024a, b).

This revision contributes taxonomic and nomenclatural corrections to the mapping programme for “Atlas Florae Europaeae”.

## Material and methods

This study is based on herbarium specimens, examined by traditional morphological method. The diagnostic characters used in this study are the same as in Cristofolini (1991) and Sennikov and Tikhomirov (2024a).

The synonymy is based on our examination of original material available through online resources (JSTOR, JACQ) and protologues. Type designations follow the latest rules of botanical nomenclature (Turland et al. 2018). New typifications are illustrated by scanned images of herbarium specimens.

Species descriptions are omitted. Instead, diagnostic characters are discussed and comparison tables are provided for species groups.

Country-level species distributions are compiled from reliable literature and accessible herbarium specimens (B, BR, H, JE, K, L, LE, LY, MA, MW, PRC, RB, U, W, WU), which were examined largely online as scanned images via JSTOR (https://www.jstor.org) and JACQ Virtual Herbaria (https://www.jacq.org). We also used human observations documented by photographs, which were available online via iNaturalist (https://www.inaturalist.org/). The distributions in the Balkans may be incomplete due to insufficient level of local studies and limited availability of herbarium material. Some species with critically revised circumscriptions are mapped. The list of specimens or observations examined and used in mapping is made available through Internet Archive (Tikhomirov and Sennikov 2023).

## Results

### *Cytisushirsutus* group

**Taxonomy.** The diagnostic character of this species group is long patent (horizontally spreading) stiff hairs on calyces and pedicels. This group requires a thorough revision on the account of its high morphological variability. In our notes, we concentrate on selected species whose type material is known to us.

#### 1. Cytisus hirsutus

Taxon classificationPlantaeFabalesFabaceae

L., Sp. Pl. 2: 739 (1753)

480C8B70-8A95-5525-9CA9-1D81045BAD8D

**Figure 1.**
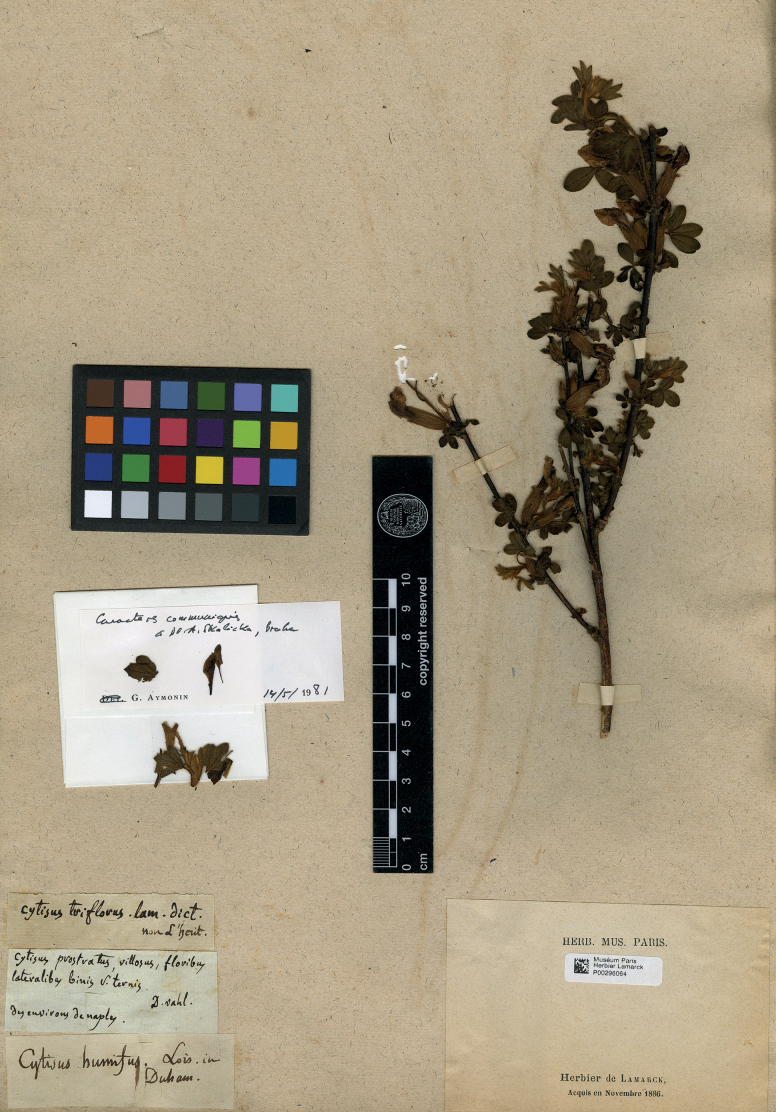
Holotype of *Cytisustriflorus* Lam.

**Figure 2.**
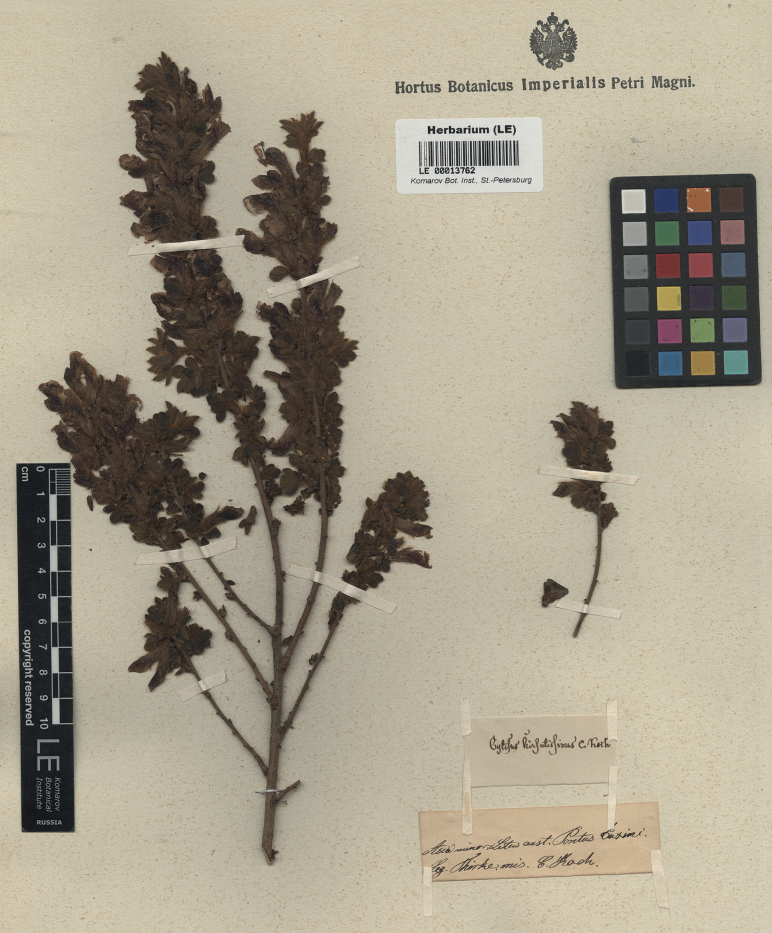
Lectotype of *Cytisushirsutissimus* K.Koch.

**Figure 3.**
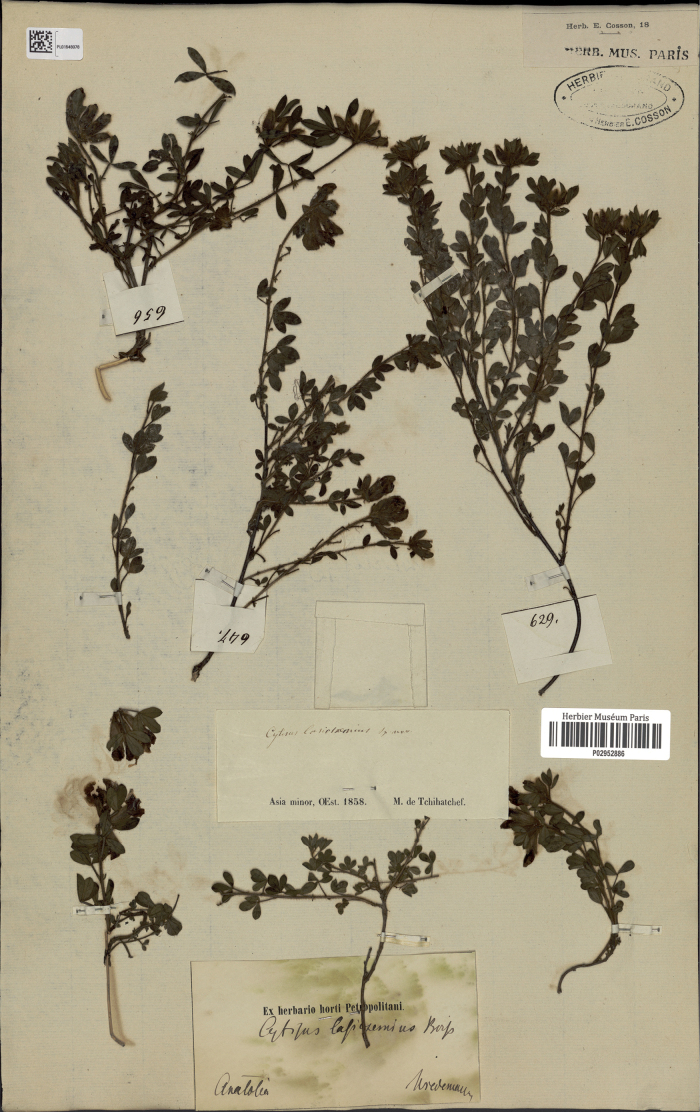
Lectotype of *Cytisuslasiosemius* Boiss (Tchihatcheff 629).

= Cytisus supinus L., Sp. Pl. 2: 740 (1753). Type. [icon] Cytisus VII in Clusius, Rar. Pl. Hist.: 96 (1601) (lectotype designated by Cristofolini and Jarvis (1991: 498)).
= Cytisus triflorus Lam., Encycl. 2(1): 250. 1786, syn. nov. – Chamaecytisustriflorus (Lam.) Skalická in Preslia 58: 23 (1986). Type. Italy. “Des environs de Naple”, [1785], *M. Vahl* in Herb. Lamarck (holotype P). Fig. 1.
= Cytisus pubescens Gilib. in Usteri, Del. Opusc. Bot. 2: 365 (1793), syn. nov. Type. [icon] Cytisus VII in Clusius, Rar. Pl. Hist.: 96 (1601) (lectotype designated here).
= Cytisus falcatus Waldst. & Kit., Descr. Icon. Pl. Hung. 3: 264, t. 238 (1812) – Chamaecytisusfalcatus (Waldst. & Kit.) Holub in Folia Geobot. Phytotax. 18(2): 204 (1983) – Chamaecytisustriflorussubsp.falcatus (Waldst. & Kit.) Pifkó in Stud. Bot. Hung. 38: 13 (2007). Type. Croatia. “In alpe Plissivicza et in monte Merszin”, *P. Kitaibel* in Herb. Kitaibel XXIV: 170 (lectotype BP, designated by Kováts (1992: 40)).
= Cytisus hirsutissimus K.Koch, Linnaea 19(1): 62 (1846) – Cytisushirsutusvar.hirsutissimus (K.Koch) Boiss., Fl. Orient. 2: 51 (1872) – Chamaecytisushirsutussubsp.hirsutissimus (K.Koch) Ponert in Feddes Repert. 83(9–10): 619 (1973) – Chamaecytisushirsutissimus (K.Koch) Czerep., Sosud. Rast. SSSR: 229 (1981). Type. Turkey. Trabzon Province: “Litus australis Pontus Euxini”, [1843], *Thirke* (lectotype LE 00013762, designated here; isolectotype LE). Fig. 2.
= Cytisus lasiosemius Boiss. in Tchihatcheff, Asie Min., Bot. 1: 12 (1860), syn. nov. – Chamaecytisuslasiosemius (Boiss.) Pifkó in Barina, Distrib. Atlas Vasc. Pl. Albania: 466 (2017) – Chamaecytisusheuffeliisubsp.lasiosemius (Velen.) Niketić in Bull. Nat. Hist. Mus. Belgrade 14: 84 (2021). Type. Turkey. “Asia Minor, OEst, 1858” [= between Samsun and Tekkeköy], 1858, *P.A. Tchihatcheff 629* (lectotype P 02952886, designated here). Fig. 3.
= Cytisus falcatus subsp. albanicus Degen & Dörfl. in Denkschr. Kaiserl. Akad. Wiss., Wien. Math.-Naturwiss. Kl. 64: 717 (1897), syn. nov. – Chamaecytisustriflorusvar.albanicus (Degen & Dörfl.) Micevski, Fl. Republ. Makedonija 1(5): 1135 (2001). Type. North Macedonia. “In locis humosis ad Neresi prope Üsküb [Skopje]”, 02.05.1893, *I. Dörfler 126* (syntype WU 068283).

##### Type.

Italy. Sassari: Olbia (“Prope Olbyam in Galloprovincia”), Herb. Burser XXII: 5 (lectotype UPS, designated by Cristofolini and Jarvis (1991: 498)).

##### Taxonomy.

This species has dimorphic inflorescences (Cristofolini 1991) and leaves densely hairy above. Cristofolini (1991) included various glabrescent forms into this species, which we prefer to exclude because such forms are not parts of the infraspecific variability in the material that we have examined.

##### Distribution.

Europe: mountain areas from western France to the Eastern Carpathians longitudinally, from southern Poland to southern Italy latitudinally (Cristofolini 1991; Cristofolini and Troía 2017).

##### Notes on nomenclature.

In the protologue of *Cytisussupinus*, Linnaeus (1753) cited three synonyms borrowed from Clusius (1601), of which one synonym (“*Cytisus* VII. species altera Clus. hist. 1. p. 96”) was cited twice. This erratic way of citation evokes the idea of corrupted references. We checked these double-cited references against the relevant synonyms in Bauhin (1671), which were linked with Clusius (1601) by Linnaeus (1753) and in the earlier treatments of Clusius (1583). The first instance of this reference, cited by Linnaeus (1753), belongs to *Cytisi* VII. species altera (Clusius 1601: 97), which is not accompanied by any illustration. The second citation actually refers to *Cytisus* VII (Clusius 1601: 96) with an illustration, which was designated by Cristofolini and Jarvis (1991: 498) as a lectotype of *C.supinus*. Although Cristofolini and Jarvis (1991) cited *Cytisus* VII. species altera as the lectotype, they unambiguously referred to the same illustration as Linnaeus, thus making the same technical citation error. We provide a correct citation here.

The protologue of *Cytisustriflorus* was based on the only cited specimen collected by Martin Vahl in Naples in 1785 (collection date from Lanzoni (1930)). This specimen was designated as a lectotype by Skalická (1986), but is most likely the holotype.

The species name *Cytisustriflorus* was misfortunately resurrected from oblivion by Skalická (1986) and accepted by Cristofolini (1991) for a segregate of *C.ratisbonensis* s.l., which is superficially similar to and often confused with *C.hirsutus*. Skalická (1986) examined the type specimen of this species name on the basis of a photograph which apparently did not show its features of pubescence. We requested a high-quality scanned image of the type from P-Lam; its examination revealed that the calyces, pedicels and petioles of this plant are covered by long upright setose hairs, which do not cover the plant tissues. These hairs are clearly distinct from the subappressed pubescence of dense thin hairs in the *C.ratisbonensis* group, which completely covers the plant parts, and correspond to the characters of *C.hirsutus*. Since the usage of this plant name after Skalická (1986) is relatively new and unstable (e.g. in Eastern Europe, the name *C.lindemannii* is still used for this species: Czerepanov (1995), Fedoronchuk (2019)) and the taxonomy of the *C.ratisbonensis* group has been in flux, the disappearance of this species name will not be of principal inconvenience for the users of plant nomenclature.

*Cytisuspubescens* Gilib. was originally introduced in Gilibert (1782), which is included in the list of suppressed works, thus disavowing valid publication of all new names of species and infraspecific taxa published in this book. This species name was validly published in a revised version of the same book (Gilibert 1793) which was reprinted from its original, also suppressed edition (Gilibert 1785). Since the reprint was not explicitly suppressed, its species plant names are considered validly published and may compete for priority (e.g. Ardenghi (2015)).

There are no extant herbarium specimens associated with the protologue of *C.pubescens* (Shiyan et al. 2013). The only element of its original material in existence is an illustration cited in the protologue, *Cytisus* VII (Clusius 1601: 96). This illustration is drawn from plants occurring in Spain (“praesertim Baetica”; this Roman Province largely corresponds to Andalucia) and represents *C.hirsutus* (Cristofolini and Jarvis 1991). Although Gilibert (1793) clearly described a plant of the *C.ratisbonensis* group under his *C.pubescens*, the illustration cited in the protologue mandates the reduction of this species name to a synonym of *C.hirsutus*, which is formally effected here by lectotypification.

*Cytisusfalcatus* was described as a relative of *C.hitsutus* (Waldstein & Kitaibel, 1812). Its pods are hairy and leaflets are sparsely hairy above, thus indicating the synonymy with *C.hirsutus* rather than *C.ciliatus* as treated by Micevski (2001) and Pifkó (2005). Cristofolini (1991) erroneously added *C.falcatus* to the synonymy of *C.triflorus* (which was a member of the *C.ratisbonensis* group in his sense).

The main collection of K.Koch was acquired to B in 1913 (Ulbrich 1917) and subsequently destroyed with few exceptions (Lack 1978). The specimens of *Cytisus* described by Koch survived at LE only (Edmondson and Lack 1977), and this material is designated as a lectotype of *C.hirsutissimus* here. Thirke labelled his collections with very generic designations. but Koch (1846) recorded that Thirke’s collecting activities took place around Trabzon and, to a lesser extent, Samsun in 1843.

We traced two specimens from the original collection of *C.hirsutissimus* at LE. As the protologue states that calyces of this species are covered by horizontally spreading hairs (Koch 1846), thus corresponding to the diagnostic characters of *C.hirsutus*, we designate a specimen (LE 00013762) whose characters are in complete agreement with the protologue.

Some authors (Kreczetowicz 1940; Grossheim 1952; Portenier and Solodko 2002) treated *C.hirsutissimus* as endemic to the Caucasus, which reportedly differed from the East European *C.lindemannii* (= *C.elongatus*) in longer pedicels and a patent (vs. subappressed) pubescence of the whole plant. These minor and variable characters cannot be considered species-specific, and *C.hirsutissimus* of these authors was correctly identified with *C.triflorus* (Cristofolini 1991). Gibbs (1970) placed *C.hirsutissimus* in the synonymy of *C.hirsutus* on account of its lateral inflorescences (his treatment maintained the difference between *C.hirsutus* and *C.supinus*, thus artificially dividing a single species with dimorphic inflorescences, whereas *C.triflorus* is a species with monomorphic lateral inflorescences). Our designated lectotype confirms the latter synonymisation.

*Cytisuslasiosemius* Boiss. was described from Asiatic Turkey (“inter Samsun et Tekekoi [Tekkeköy]”, now Bayraktepe National Park, Samsun Province). In the protologue, Boissier (Tchihatcheff 1860) compared the new species with *C.supinus* (= *C.hirsutus*), and distinguished it from the latter by acute leaflets and hairy standard. These characters are variable within *C.hirsutus*, and Gibbs (1970) rightly placed *C.lasiosemius* to the synonymy of his *C.supinus*. On the contrary, Cristofolini (1991) accepted *C.lasiosemius* as a priority name for *C.frivaldszkyanus* Degen, which also has rather patent hairs. This treatment cannot be accepted because the pubescence of *C.lasiosemius* is composed of long, sparsely situated horizontal hairs on its stems, petioles and pedicels, typical of *C.hirsutus*, whereas the pubescence of *C.frivaldszkyanus* is very densely covering the stems, petioles and pedicels and consists of both long and short curved hairs, like in the *C.ratisbonensis* group (Sennikov and Tikhomirov 2024a). We confirm the opinion of Gibbs (1970) and add *C.lasiosemius* to the synonymy of *C.hirsutus*.

The original material of *C.lasiosemius* consists of a few specimens collected by P.A. Tchihatcheff in Turkey during 1858 (Tchihatcheff 1860). These specimens are accompanied by tiny field tickets with different field numbers, thus indicating that they are different gatherings. Niketić (2021) designated a complete herbarium sheet at P with three gatherings as a lectotype, which is inadmissible. We restrict this choice to a single gathering numbered 629.

#### 1a. Cytisus hirsutus var. ciliatus

Taxon classificationPlantaeFabalesFabaceae

(Wahlenb.) Hazsl. in Verh. K.K. Zool.-Bot. Ges. Wien 1: 201 (1852)

A76A0B62-3B2E-5E5D-AEB5-81E7A8CD05B2

– Cytisus ciliatus Wahlenb., Fl. Carp.: 219 (1814) – Cytisusprostratusvar.ciliatus (Wahlenb.) W.D.J.Koch, Syn. Deut. Schweiz. Fl. 1: 155 (1837) – Cytisushirsutussubsp.ciliatus (Wahlenb.) Simonk. in Math. Term. Közlem. 22: 376 (1888) – Chamaecytisustriflorussubsp.ciliatus (Wahlenb.) Holub in Bertová, Fl. Slovenska IV(4): 38 (1988).
= Cytisus glaber L.f., Suppl. Pl.: 328. 1782, non Lam. 1779, nom. illeg. (Art. 53.1) – Chamaecytisusglaber Rothm. in Feddes Repert. Spec. Nov. Regni Veg. 53: 143 (1944). Type. Not designated.
= Cytisus serotinus Kit. ex DC., Prodr. 2: 156 (1825) – Cytisushirsutusvar.serotinus (Kit. ex DC.) Soó in Veröff. Geobot. Inst. Rübel Zürich 6: 254 (1930). Type. Western Ukraine (Mukachevo) or Romania (Satu Mare). Locality unknown, 1815, *P. Kitaibel* (holotype G-DC barcode G00477721; isotypes BM barcode BM000750883, M barcode M0210789).

##### Type.

**Figure 4.**
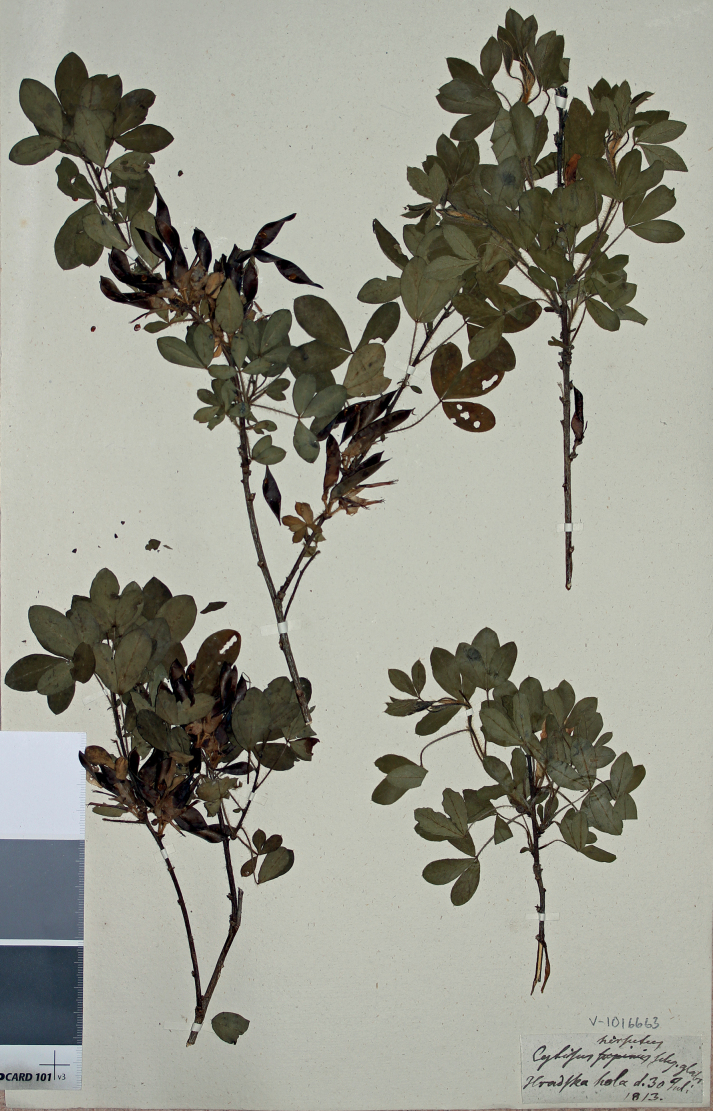
Lectotype of *Cytisusciliatus* Wahlenb.

Slovakia. Žilinský kraj: “Hradska hola” [Hradská Hora], 30.07.1813, *G. Wahlenberg* (lectotype UPS V-1016663, designated here). Fig. 4.

##### Distribution.

Europe: certainly present in Slovakia, Ukraine, Hungary, Romania and the Balkans; reported as “*C.falcatus*” from North Macedonia (Micevski 2001).

##### Notes on taxonomy and distribution.

This taxon was described from the vicinities of Liptovský Hrádok in present-day Slovakia (Wahlenberg 1814) and occurs in the mountains surrounding the Pannonian Plain and in the Balkans (Holub and Bertová 1988; Pifkó 2009 and our data). *Cytisusciliatus* is closely related to *C.hirsutus*, but differs from the latter by the upper side of its leaf laminae and by pod surfaces being glabrous or nearly so (vs. regularly hairy). So far, we have no evidence that the distribution of hairy and glabrous plants of *C.hirsutus* is separate; this distinction denotes the same casual loss of pubescence as observed in some other species of *Cytisus* (C.ruthenicusvar.zingeri Nenjukov: Sennikov et al. (2021); C.polytrichusvar.subglabratus Val.N.Tikhom. & Sennikov, see below) and corresponds to the rank of variety.

Some authors (Bernard 1977) interpreted the name *Cytisusglaber* as corresponding to *C.hirsutus*, which cannot be true because of its leaves glabrous above. Judging from the glabrous leaves of the plant and its occurrence in “Austria”, *C.glaber* is an earlier (albeit illegitimate and therefore unusable) synonym of *C.ciliatus* Wahlenb. (*C.hirsutus* s.l.). Tzvelev (1987) formally accepted *Chamaecytisusglaber* (with *C.elongatus* mis-added to its synonymy) and applied it to west Ukrainian and cultivated plants of Central European origin with erect stems, leaves glabrous above, lateral inflorescences and patent pubescence, which agrees with our interpretation.

*Cytisusserotinus* is a plant with the leaves glabrous above, which belongs to the *C.hirsutus* group. It was originally recognised due to its presumed late flowering season, but merely coincides with *C.ciliatus*.

##### Notes on nomenclature.

Wahlenberg (1814) distinguished *Cytisusciliatus* from *C.hirsutus*, which was the original name for his material, by the pubescence of its leaves and pods. In the collections of UPS, where the Herbarium of Wahlenberg is housed, two specimens of the original material were found, both corresponding to the original description and the provenance cited in the protologue. One specimen bears precise collection data, but the draft name of the taxon (*C.hirsutus* [...] glabris) written by Wahlenberg, whereas the second specimen bears the final plant name (*C.ciliatus*), but generalised collection data (“e montibus Carpaticis”) written by C.P. Thunberg. As both specimens correspond to the taxon as circumscribed by Wahlenberg and are undoubtedly linked with the protologue, we prefer the specimen with exact provenance from the author’s collection as a lectotype.

Despite all searches, we were not able to trace any herbarium material linked with the protologue of *C.glaber* (Linnaeus filius 1782), in which a species with the leaves glabrous above and slightly hairy below was described from “Austria”. The only original element, an illustration of “Cytisusglaber, siliqua angusta” in Bauhin and Cherler (1650: 373) was rejected by Cristofolini (1991) as conflicting with the original description (calyces depicted as campanulate, whereas the protologue stated the calyx being “oblongus subventricosus”), although this presumed conflict may be explained by the crude nature of this drawing. So far, this species name remains untypified and interpreted on the basis of the protologue (Tzvelev 1987).

A later synonym belonging to the same taxon is *C.serotinus* Kit. ex DC. (Candolle 1825), described from historical “Hungary” without a further specification. Pifkó (2005) designated a lectotype at BP; since no specimens were cited by Candolle as syntypes, his only specimen used for the original description is the holotype, and the lectotype at BP has no standing. The only original specimen in Candolle’s herbarium at G is lacking a precise provenance, which can be derived from comparisons with the main collections of P. Kitaibel kept at BP (Jávorka 1957) and from the diaries of Kitaibel (Gombocz 1945; Lőkös 2001).

Three specimens identified as *C.serotinus* are preserved in the herbarium of Kitaibel at BP (Pifkó 2005), collected near Mukachevo in present-day Ukraine and at Gödöllő in present-day Hungary. Kitaibel (Lőkös 2001) also mentioned that he collected this species near Szatmár (now Satu Mare in Romania, near the border with Hungary and Ukraine). The specimen at G-DC is dated as received in 1815 and seemingly was collected during that year on the way from Mukachevo to Satu Mare (Lőkös 2001).

#### 2. Cytisus polytrichus

Taxon classificationPlantaeFabalesFabaceae

M.Bieb., Fl. Taur.-Caucas. 3: 477 (1819)

ED950FF8-986D-5431-89E8-2247E4265E1D

– Cytisus hirsutus var. polytrichus (M.Bieb.) Briq., Étud. Cytises Alpes Mar.: 171 (1894) – Cytisushirsutussubsp.polytrichus (M.Bieb.) Hayek in Repert. Spec. Nov. Regni Veg. Beih. 30(1): 898 (1926) – Chamaecytisuspolytrichus (M.Bieb.) Rothm. in Feddes Repert. Spec. Nov. Regni Veg. 53: 144 (1944) – Chamaecytisushirsutussubsp.polytrichus (M.Bieb.) Ponert in Feddes Repert. 83: 619 (1973).
= Cytisus demissus Boiss., Fl. Orient. 2: 54 (1872) – Cytisushirsutusvar.demissus (Boiss.) Halácsy, Consp. Fl. Graec. 1: 337 (1900) – Chamaecytisuspolytrichusvar.demissus (Boiss.) Kuzmanov in Jordanov, Fl. Narodna Republ. Bulg. 6: 82 (1976). Type. Greece. “In Olymp. Thessaliae”, *P. Aucher-Éloy 1111* (holotype G; isotypes BM 000750882, K 000829496, MPU 023084).

##### Type.

Crimea. “Taur. merid.”, Herb. Bieberstein (lectotype LE 01080952, designated by Krytzka et al. (1999: 611)).

##### Distribution.

Europe: France, Italy, Balkans, Greece, Crimea (Cristofolini 1991); Asia: Russian Western Caucasus.

##### Notes on taxonomy and distribution.

*Cytisuspolytrichus* sharply differs from *C.hirsutus* in its creeping stems, small leaves and constantly axillar flowers (Cristofolini 1991).

Plants of this species have been known from the Western Caucasus under a wrong name, *C.wulffii* auct. (Kreczetowicz 1940; Grossheim 1952). The latter species is endemic to the Crimea and differs from *C.polytrichus* in appressed (vs. strictly patent) hairs on its leaves and calyces (Sennikov and Tikhomirov 2024a).

##### Notes on nomenclature.

Krytzka et al. (1999) designated the only suitable specimen at LE as lectotype, following the unpublished annotation by N.N. Tzvelev.

#### 2a. Cytisus polytrichus var. subglabratus

Taxon classificationPlantaeFabalesFabaceae

Val.N.Tikhom. & Sennikov var. nov.

C93126AE-1E6E-563E-9C29-7105DEF0BA16

urn:lsid:ipni.org:names:77336842-1

##### Type.

Russia. Krasnodar Region: Krasnaya Poliana, Chugush Mt., Osmanova Poliana, alt. 2140 m, rocky subalpine meadows, 11.07.1982, *E. Mordak 1920* (holotype LE 01070725).

##### Diagnosis.

Leaves and young branches subglabrous.

##### Distribution.

Asia: Russian Western Caucasus. So far, known from the holotype.

##### Notes on taxonomy and distribution.

Plants of this variety were found within the same distribution area as the type variety, thus indicating infrapopulation variability.

### *Cytisusaustriacus* group

Table 1

**Table 1.** Diagnostic characters in the *Cytisusaustriacus* group.

	*C.absinthioides*	*C.austriacus*	*C.frivaldszkyanus*	*C.jankae*	*C.calcareus*
stems	tall (30–60 cm), erect, hairs 0.3–0.6 mm long, appressed, sericeous	tall (20–50(70) cm), erect, hairs 1.5–2.5 mm long, appressed	low (10–30 cm), ascending, hairs 1.5–2.0 mm long, subpatent	low (10–20 cm), ascending, hairs (0.7–)1.0–2.0 mm long, laxly appressed	low (10–40 cm), ascending, hairs (0.7–)1.0–2.0 mm long, laxly appressed
leaves	leaflets narrowly lanceolate, acute, hairs 0.3–0.6 mm long, appressed, sericeous	narrowly lanceolate to lanceolate, acute, hairs 1.5–2.5 mm long, appressed	leaflets elliptic-lanceolate to obovate, broadly acute, hairs 0.8–1.5 mm long, subpatent	lanceolate or slightly oblanceolate, acute, hairs (0.5–)0.8–1.5 mm long, appressed	leaflets elliptic-lanceolate to obovate, broadly acute, hairs (0.5–)0.8–1.5 mm long, appressed
pedicels	hairs 0.3–0.6 mm long, appressed	hairs 1–2 mm long, laxly appressed	hairs 1.0–2.0 mm long, subpatent to patent	hairs 1.0–2.0 mm long, laxly appressed	hairs 1.0–2.0 mm long, laxly appressed to subpatent
calyx	7–9 mm long, hairs 0.3–0.8 mm long, appressed	10–13 mm long, hairs 1–2.5 mm long, laxly appressed to subpatent	10–12 mm long, hairs 1.3–2.5 mm long, subpatent to patent	(8–)10–13 mm long, hairs 1.0–2.2 mm long, laxly appressed	10–13 mm long, hairs 1.5–2.5 mm long, laxly appressed to subpatent
pods	hairs appressed	hairs appressed	hairs patent	hairs appressed	hairs appressed to subpatent

**Taxonomy.** The diagnostic characters of this species group are erect stems, dense capitate inflorescences and long thin silky hairs on calyces and pedicels. The knowledge on this group is highly incomplete, especially regarding the variability of *Cytisusaustriacus* L. s.l.

#### 3. Cytisus austriacus

Taxon classificationPlantaeFabalesFabaceae

L., Sp. Pl., ed. 2, 2: 1042 (1763)

53534D22-4BD0-5405-9E69-CB18A754FF44

**Figure 5.**
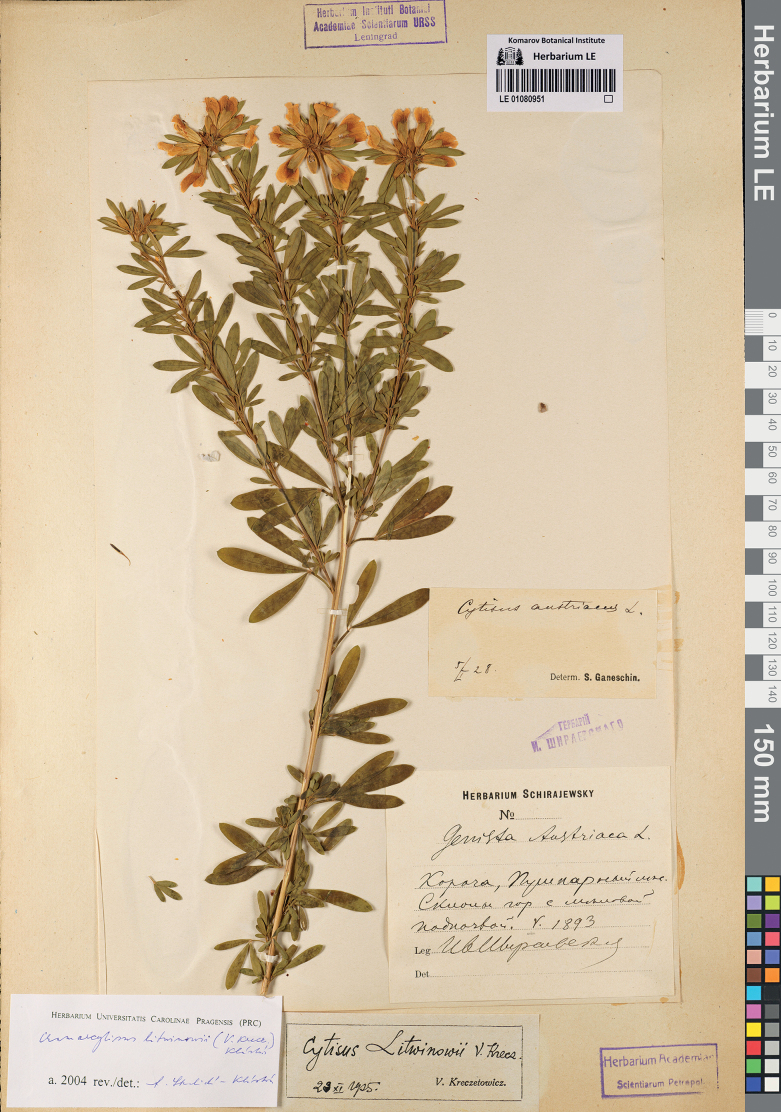
Holotype of *Cytisuslitwinowii* V.I.Krecz.

– Chamaecytisus austriacus (L.) Link, Handb. 2: 155 (1831).
= Cytisus supinus var. noeanus Briq., Étud. Cytises Alpes Mar.: 182 (1894) – Cytisusaustriacussubsp.noeanus (Briq.) Jáv., Magyar Fl. 2: 608 (1924). Type. Greece. “Rumelia” [Nicopolis], 06.1846, *Noe* [251] (syntype K 000829490).
= Cytisus litwinowii V.I.Krecz. in Bot. Zhurn. SSSR 25: 256 (1940), syn. nov. – Chamaecytisuslitwinowii (V.I.Krecz.) Klásk. in Preslia 30: 214 (1958). Type. Russia. Belgorod Region: Korocha Town, “Pushkarnoe forest” [west of Pushkarnoe Village], hills, on calcareous substrate, 05.1893, *I. Schirajewsky* (holotype LE 01080951). Fig. 5.
= Chamaecytisus pseudojankae Pifkó & Barina in Stud. Bot. Hung. 47(1): 169 (2016), syn. nov. Type. Albania. District of Korçë (Rrethi i Korçës), Thatë Mountains (Mali i Thatë), ca 1.7 km north of village “Zvezdë”, on the south-eastern ridge of Mount “Zvezdë” (1,833 m), in rocky grassland, on limestone, 40.74774°N, 20.86148°E, 1477 m elev., 25.05.2007, *Z. Barina, D. Pifkó & Cs. Németh 11736* (holotype BP 750418; isotype W 2010-03241).

##### Type.

Historical Hungary (“Ungaria”). Herb. Burser XXII: 3, left-hand specimen (lectotype UPS, designated by Cristofolini in Turland and Jarvis (1997: 468)).

##### Distribution.

Europe: mountainous regions from Austria to western Ukraine and from southern Poland to Greece and European Turkey, with the presence in southern East European uplands; Asia: Turkey, Russian Caucasus (Gibbs 1970; Tzvelev 1987; Cristofolini 1991).

##### Notes on taxonomy.

This species is highly variable in respect of the pubescence on its leaves and calyces and is currently recognised in a broad sense, with some infraspecific taxa (Cristofolini 1991). Our current treatment is focused on the typical plants, corresponding to *C.austriacus* s. str.

A short-leaved variant of the species was separated as C.austriacussubsp.microphyllus “(Boiss.) Boiss.” by Cristofolini (1991), probably because of *Baldacci 315* (BM 000750880) which was the basis for the treatment of C.austriacusvar.microphyllus in Baldacci (1899). This collection from Mt. Smolikas in north-western Greece consists of subalpine plants of *C.austriacus* s. str. which have regrown after damage and developed smaller leaves, otherwise being in agreement with the type.

##### Notes on nomenclature.

*Cytisuslitwinowii* V.I.Krecz. was described as a local endemic of the Central Russian Upland, confined to calcareous substrates (Kreczetowicz 1940). This plant was originally distinguished because of its lesser developed pubescence and golden-yellow flowers, which are smaller than in *C.blockii* V.I.Krecz. (= *C.kerneri* Błocki). Another reason to distinguish this plant as a separate taxon was its confinement to the area of presumably relic pine forests and shrublands of the steppe area of Central European Russia, which reportedly harboured endemic taxa of Tertiary age (Kozo-Polansky 1931). However, this area of endemism has been confuted by other researchers, who considered its age being early postglacial and its relics being taxonomically indistinct (Grosset 1964). Among the presumed endemics of this territory, *Daphnejulia* K.-Pol. turned out to be a synonym of *D.cneorum* L. (Grosset 1964) and *Tanacetumalaunicum* K.-Pol. was synonymised with *Chrysanthemumzawadskii* Herbich (Tzvelev 1994), whereas *Cotoneasteralaunicus* Golitsin appeared to be a synonym of *C.integerrimus* Medik. (Sennikov 2011).

Further authors (Heywood and Frodin 1968; Tzvelev 1987) accepted *C.litwinowii* and distinguished it from *C.austriacus*, which also occurs in Central European Russia, by its leaflets glabrous or very poorly (sparsely) pubescent above (vs. densely appressed-hairy in *C.austriacus*). Following these authorities, *C.litwinowii* was accepted in major compilations (Yakovlev et al. 1996; Govaerts et al. 2021).

We examined the holotype of *C.litwinowii* at LE and realised that the leaflets of this plant, which had grown in the shade, are regularly pubescent above, but the hairs are poorly recognisable due to overpressing. As pubescence of leaflets was the main diagnostic characters for *C.litwinowii* and no other material of the taxon is known, but the holotype, we reduce it to a synonym of *C.austriacus*. The placement of *C.litwinowii* in the synonymy of *C.blockianus* Pawł. (Cristofolini 1991), which was accepted by some databases (Roskov et al. 2006), cannot stand because the latter species does not occur east of the Carpathians (Tzvelev 1987). Besides, the bright flower colour of *C.litwinowii* agrees particularly with the characters of *C.austriacus*, rather than the pale flower colour of *C.blockianus* (Tzvelev 1987).

Pifkó and Barina (2016) described *C.pseudojankae* Pifkó & Barina as a strongly branching plant with undeveloped axillar shoots, small, narrowly lanceolate leaflets and laxly appressed pubescence, which they compared with the *C.austriacus* aggr., but placed in the *C.eriocarpus* aggr. Such plants were considered endemic to a restricted area near Lake Prespa at the borders of Albania, North Macedonia and Greece (Pifkó and Barina 2016; Bergmeier et al. 2020). According to the description and drawing of *C.pseudojankae* in Pifkó and Barina (2016), this taxon is very similar to *C.austriacus* in its strong and upright stems (vs. weak and ascending stems in *C.eriocarpus* s.l.), habit and narrowly lanceolate leaf shape.

The original material of *C.pseudojankae* (Pifkó and Barina 2016) consists of plants superficially looking like having lateral flowers; however, these plants are typical members of the *C.austriacus* group with capitate inflorescences, and the seemingly lateral flowers observed in *C.pseudojankae* are a result of its abundant branching, with the uppermost branches, much abbreviated, going to flower and thereby forming a pseudolateral inflorescence. Their leaves are similar to those of the plants treated as C.austriacussubsp.microphyllus by Cristofolini (1991).

#### 4. Cytisus jankae

Taxon classificationPlantaeFabalesFabaceae

Velen. in Abh. Königl. Böhm. Ges. Wiss. 1889: 31 (1890)

2EF95617-4C92-5066-B670-C41C028A56B8

– Chamaecytisus jankae (Velen.) Rothm. in Feddes Repert. 53: 144 (1944) – Chamaecytisusheuffeliisubsp.jankae (Velen.) Niketić in Bull. Nat. Hist. Mus. Belgrade 14: 83 (2021).
= Cytisus austriacus var. pindicola Degen in Nuovo Giorn. Bot. Ital., nov. ser. 6: 152 (1899), “pindicolus”, syn. nov. – Cytisuspindicola (Degen) Halácsy, Consp. Fl. Graec. 1(2): 338 (1901). Described from a few localities in north-western Greece (syntypes K 000829489, PRC 454944, 454945, WU-Halácsy 0072806).

##### Type.

**Figure 6.**
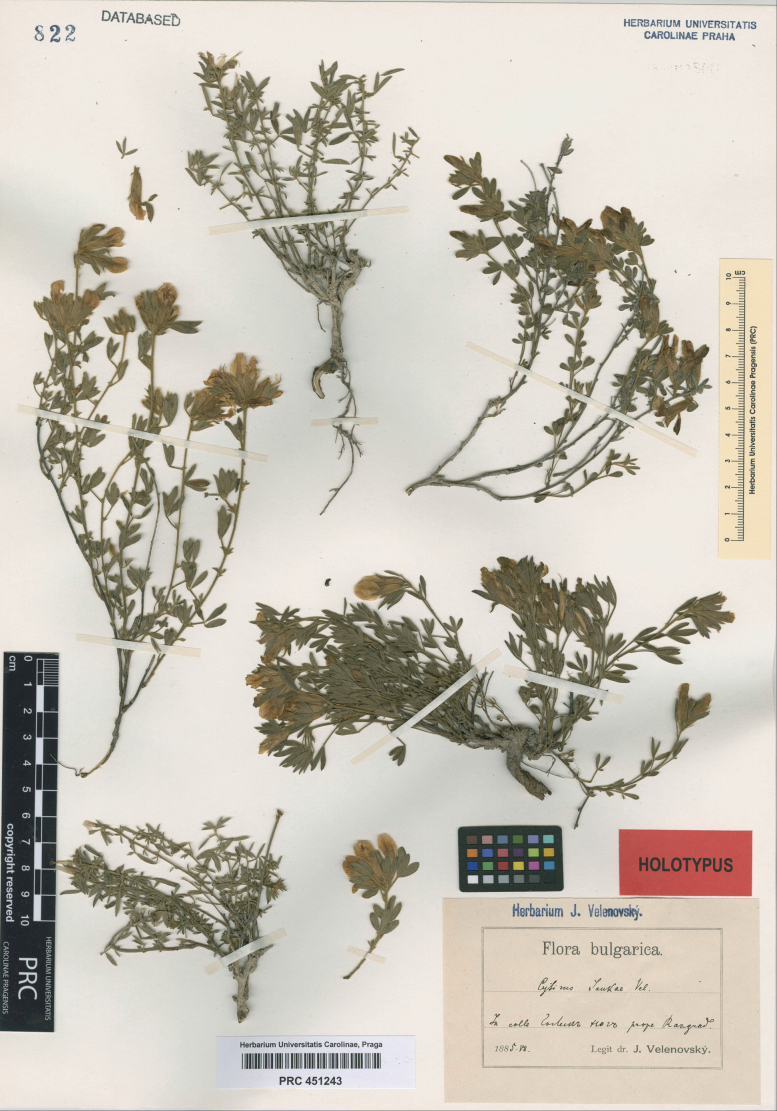
Lectotype of *Cytisusjankae* Velen. (plant above the label).

Bulgaria. Razgrad Region: “In colle Golem Jug prope Razgrad”, 07.1885, *J. Velenovský* (lectotype PRC 451243, single plant above the label, designated here). Fig. 6.

##### Distribution.

**Figure 7.**
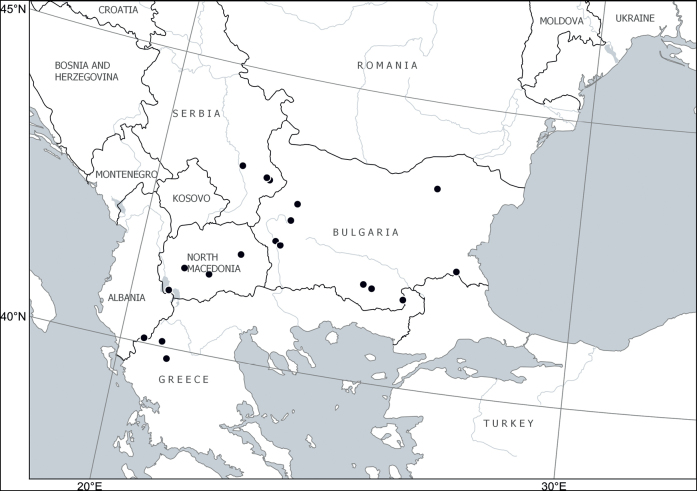
Distribution of *Cytisusjankae* Velen.

Europe: Balkan Peninsula (Albania, Bulgaria, Greece, North Macedonia, Serbia) (Diklić 1972; Kuzmanov 1976; Micevski 2001; Assyov and Petrova 2012; Barina et al. 2018; Niketić 2021). Fig. 7.

##### Notes on taxonomy.

Cristofolini (1991) placed *C.jankae* next to *C.austriacus*, thus indicating their affinity. Both species share capitate inflorescences, lanceolate leaves and rather appressed pubescence on all green parts, but *C.jankae* differs from *C.austriacus* s.str. by its constantly small size and prostrate habit. Its recent subordination to *C.heuffelii* (Niketić 2021), which differs in its calyx being 7–8 mm long (vs. 10–13 mm long in *C.jankae*), is hardly justified.

According to their original material, *C.pindicola* belongs to the synonymy of *C.jankae* as typified here. The synonymisation of *C.pindicola* with *C.frivaldszkyanus* proposed by Barina et al. (2018) is not supported by their diagnostic characters (Table 1).

##### Notes on nomenclature.

The original material of *Cytisusjankae* Velen., mounted as a single specimen (PRC 451243), is highly heterogeneous and consists of six fragments of small plants with stems ascending from woody caudices, with capitate inflorescences and narrow leaves, which are referable to three species. In spite of its apparent heterogeneity, this entire specimen has been recently designated as a lectotype of the species name (Niketić 2021).

Two linear-leaved fragments (top centre, bottom left) on this specimen belong to *C.absinthioides* Janka, which is another species of the Balkans. This species is sometimes (Cristofolini 1991; Govaerts et al. 2021) merged with *C.eriocarpus* Boiss. (syn. *C.smyrnaeus* Boiss.), which is characterised by its leaflets being broadly obovate to elliptic rather than narrowly lanceolate and is totally different in its habit and long spreading pubescence. *Cytisusabsinthioides* is characterised by typically upright, strongly branched stems, regular presence of abbreviated sterile shoots in the leaf axils, small flowers (with calyces 7–8 mm long), rather short subpatent pubescence on the stems and dense appressed pubescence of silvery appearance on the leaflets.

Two plants on the left and right sides are characterised by decumbent to ascending stems, narrowly lanceolate or oblanceolate leaflets and subpatent pubescence on stems and calyces, with less developed sterile shoots in leaf axils. These plants correspond to *C.pygmaeus* Willd., occurring in the Balkans and Turkey.

The plant mounted above the label is similar to *C.pygmaeus*, but differs from the latter in a densely appressed pubescence, the feature corresponding to the original description of *C.jankae* which reads “foliolis linearibus vel lineari-spathulatis ... calycis adpresse sericei ...” (Velenovský 1890). The small fragment alongside the label probably belongs to the same species. As this plant is in good agreement with the protologue, we designate it as a lectotype of *C.jankae*.

Other low-growing and small-leaved variants presumably belonging to the same group are *C.pseudopygmeus* Davidov and *C.georgievii* Davidov, described from the Pontic part of Bulgaria (Davidoff 1902) and synonymised with *C.jankae* by Kuzmanov (1976). We refrain from any assessment of these species names because we were not able to examine any original material.

*Cytisuspindicola* (Degen) Halácsy agrees with the type of *C.jankae*, but slightly differs from the latter in slightly shorter hairs on stems (0.7–1 mm long vs. 1–2 mm long in *C.jankae*) and leaves (0.5–0.8 mm long vs. 0.8–1.5 mm long in *C.jankae*) and in shorter calyces (8–10 mm long vs. 10–13 mm long in *C.jankae*). *Cytisuspindicola* was previously placed in a subspecies of *C.austriacus* (Cristofolini 1991, as C.austriacussubsp.microphyllus), but differs from the latter in shorter leaves and a different habit.

The original material of Cytisusaustriacusvar.pindicola Degen (Baldacci 1899) consists of four gatherings which were distributed under a single number, as *Baldacci 110*. K.I. Christensen intended to designate a lectotype at W, but the only specimen in that collection is a mixture of four indistinguishable gatherings (Reich et al. 2021). Lectotypification is advisable with Degen’s material at BP.

#### 5. Cytisus calcareus

Taxon classificationPlantaeFabalesFabaceae

(Velen.) Sennikov & Val.N.Tikhom. comb. nov.

F13771E2-09E7-5BF1-A7A9-99F29978189E

urn:lsid:ipni.org:names:77336843-1

– Cytisus pygmaeus var. calcareus Velen., Fl. Bulg. Suppl. 1: 71 (1898) – Chamaecytisuscalcareus (Velen.) Kuzmanov in Jordanov, Fl. Narodna Republ. Bulg. 6: 103 (1976).

##### Type.

Bulgaria. “Supra Belledihan in calcareis”, 05.1893, *J. Velenovský* (lectotype PRC 451952, designated by Kuzmanov (1976: 103)).

##### Distribution.

**Figure 8.**
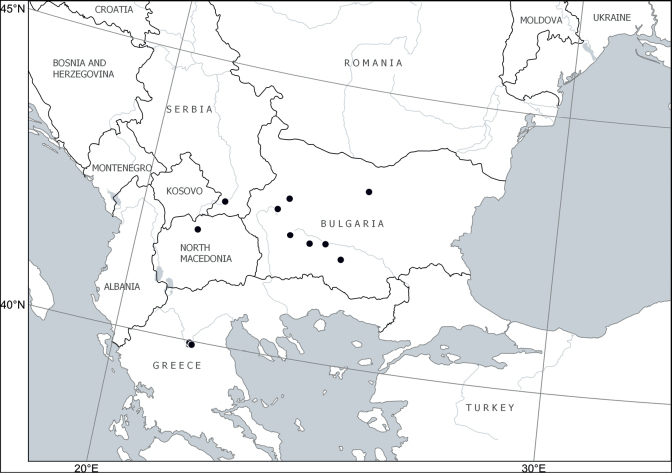
Distribution of *Cytisuscalcareus* (Velen.) Sennikov & Val.N.Tikhom.

Europe: Balkan Peninsula (Bulgaria, Greece, North Macedonia, Serbia) (Kuzmanov 1976; Assyov and Petrova 2012). The occurrences outside Bulgaria are confirmed or reported here (Fig. 8). Pifkó and Barina (2016) removed the report of *Chamaecytisuscalcareus* from Albania in favour of their *C.pseudojankae*, which we synonymise with *C.austriacus*.

##### Notes on taxonomy.

This miniature plant belongs to the *C.austriacus* group because of its terminal inflorescences, which are rather dense and surrounded by floral leaves. It differs from *C.austriacus* by its short habit, much smaller and shorter, subelliptic (vs. lanceolate) leaves, and from *C.jankae* by the same shape of leaves (although of similar size) and by subpatent (vs. appressed) pubescence of calyces. This species was omitted by Cristofolini (1991) and is currently recognised only in Bulgaria (Kuzmanov 1976; Assyov and Petrova 2012).

##### Notes on nomenclature.

Velenovský (1898) considered this taxon to be intermediate between *C.pygmaeus* and *C.austriacus*. The original material represents a mixture of *C.austriacus* (Kovarna, 08.1897, *Škorpil* (PRC)) and a taxon currently recognised as *C.calcareus* (Kuzmanov 1976). Kuzmanov (1976) designated the latter gathering as lectotype, thus fixing the application of the species name.

#### 6. Cytisus absinthioides

Taxon classificationPlantaeFabalesFabaceae

Janka in Oesterr. Bot. Z. 22: 175 (1872)

BFD9B958-1B95-5130-91B4-514758C3390C

– Chamaecytisus absinthioides (Janka) Kuzmanov in Taxon 21: 336 (1972) – Chamaecytisusheuffeliisubsp.absinthioides (Velen.) Niketić in Bull. Nat. Hist. Mus. Belgrade 14: 82 (2021).
– Cytisus eriocarpus auct.: Cristofolini (1991).
– Chamaecytisus eriocarpus auct.: Pifkó and Barina (2016); Barina et al. (2018).

##### Type.

Bulgaria. “In montibus ad radices m. Perimdagh prope Nevrekop Macedoniae orientalis”, 21.08.1871, *V. Janka* (lectotype WU 0033170, designated by Pifkó and Barina (2016: 172); isolectotypes BEOU (s. n.), BP 296809, GOET 005095, W-Reichenb 44808, WU-Halácsy).

##### Distribution.

**Figure 9.**
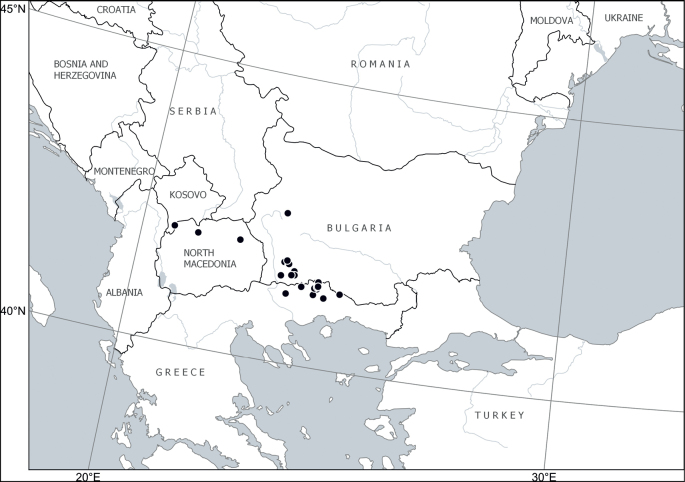
Distribution of *Cytisusabsinthioides* Janka.

Europe: Balkan Peninsula (Bulgaria, Greece, Kosovo, North Macedonia) (Diklić 1972; Kuzmanov 1976; Micevski 2001; Assyov and Petrova 2012; Niketić 2021). Fig. 9.

##### Notes on taxonomy.

*Cytisusabsinthioides* strikingly differs from any other species of the *C.austriacus* group by its habit, resembling some plants of *Artemisia* due to its tall branched stems with regularly developed sterile branches in leaf axils and dense appressed sericeous pubescence on its leaves and calyces. Its calyces and pods are distinctly small (Janka 1872).

Some recent interpretations (Cristofolini 1991) placed *C.absinthioides* to the synonymy of *C.eriocarpus*, which was treated as a broadly defined and variable species. This placement is not justified because *C.eriocarpus* clearly differs in its habit, leaf shape, subpatent pubescence and longer calyces.

Pifkó and Barina (2016) and Barina et al. (2018) reported the presence of *C.eriocarpus* in Albania, but their description matches *C.absinthioides*. The earlier records of *C.eriocarpus* in Greece (Strid 1986) employed the same taxonomic concept and should also belong to the same species (Kuzmanov 1976; Micevski 2001; Assyov and Petrova 2012).

#### 7. Cytisus frivaldszkyanus

Taxon classificationPlantaeFabalesFabaceae

Degen in Oesterr. Bot. Z. 43: 422 (1893)

77DECE20-96DD-573C-A63C-356C848830F1

**Figure 10.**
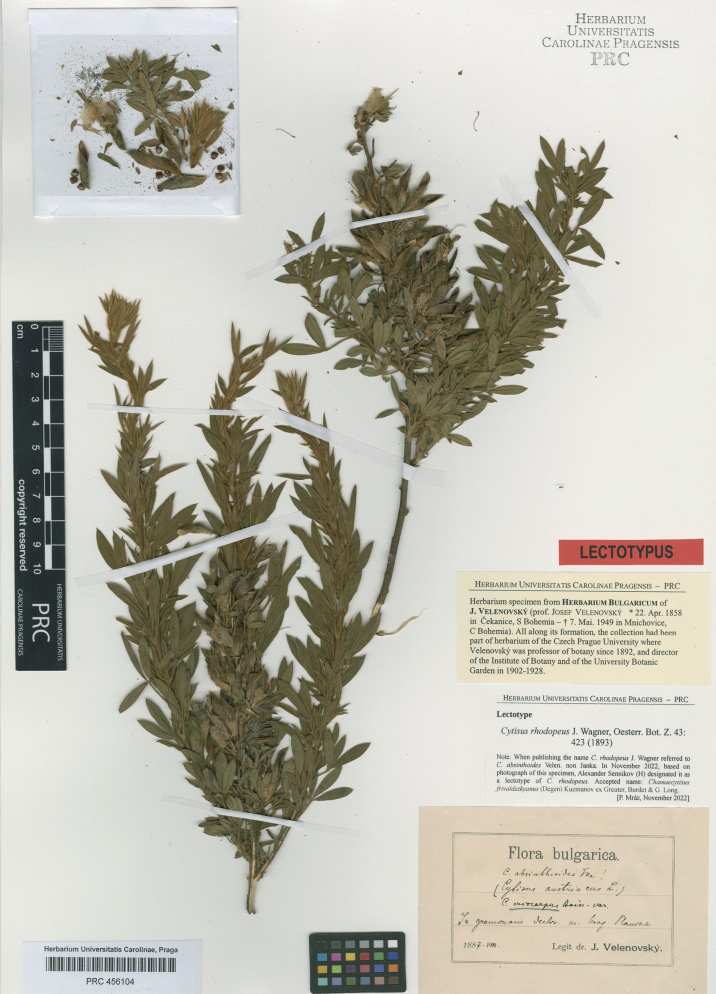
Lectotype of *Cytisusrhodopeus* J.Wagner ex Bornm.

– Chamaecytisus frivaldszkyanus (Degen) Kuzmanov in Jordanov, Fl. Narodna Republ. Bulg. 6: 110 (1976); Kuzmanov in Taxon 24: 504 (1975), comb. inval. (Art. 41.1).
= Cytisus microphyllus Boiss., Diagn. Pl. Orient., ser. 2, 2: 5 (1856), non Link (1825), nom. illeg. (Art. 53.1), syn. nov. – Cytisusaustriacusvar.microphyllus Boiss., Fl. Orient. 2: 53 (1872) – Cytisusaustriacussubsp.microphyllus (Boiss.) Cristof. in Webbia 45(2): 210 (1991). Type. Greece. “In monte Pelione”, *P. Aucher-Éloy 1109* (holotype G; isotypes BM 000750890, K 000829488).
= Cytisus rhodopeus J.Wagner ex Bornm. in Bot. Jahrb. Syst. 59(5): 465 (1925) – Chamaecytisusabsinthioidessubsp.rhodopeus (J.Wagner ex Bornm.) Kuzmanov in Taxon 21: 336 (1972), comb. inval. (Art. 41.1) – Chamaecytisusabsinthioidesvar.rhodopeus (J.Wagner ex Bornm.) Micevski, Fl. Macedon. 1(5): 1140 (2001), comb. inval. (Art. 41.1). Type. Bulgaria. “In graminosis decliv. m. Osogovska Planina”, 08.1887, *J. Velenovský* (PRC 456104, lectotype designated here). Fig. 10.
– Cytisus lasiosemius auct.: Cristofolini (1991).
– Chamaecytisus supinus subsp. lasiosemius auct.: Niketić (2021).

##### Type.

Bulgaria. “In declivibus dumetosis montis Rhodopes centralis pr. Stanimak (inter Hvojna et Bačkova)”, 06.1892, *J. Wagner 39* (syntypes JE, PRC); “In declivibus dumetosis prope Slivno (Balkan)”, 07.1893, *J. Wagner* (syntypes JE, PRC); “In dumetosis montis “Čatal Kaje” prope Slivno”, 21.07.1893, *J. Wagner 31* (syntype PRC); “Bela Cerkva”, *Skorpil* (syntype not traced).

##### Distribution.

**Figure 11.**
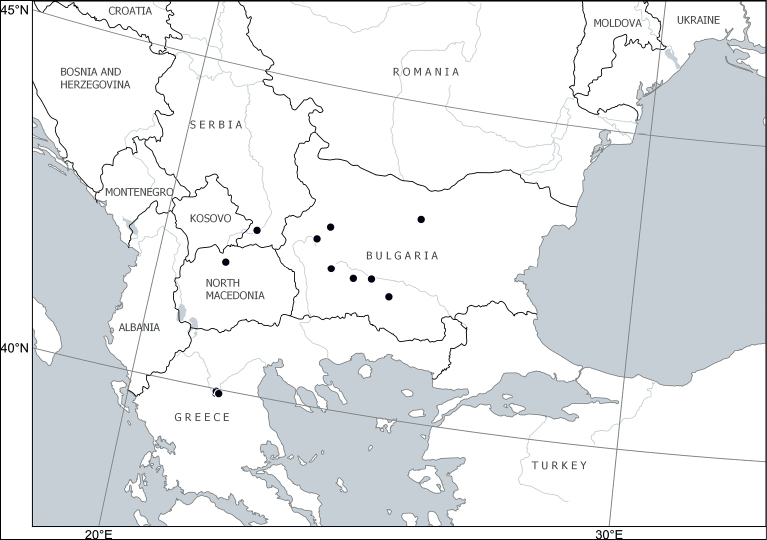
Distribution of *Cytisusfrivaldszkyanus* Degen.

Europe: Balkan Peninsula (Bulgaria, Greece, North Macedonia, Serbia) (Kuzmanov 1976; Micevski 2001; Assyov and Petrova 2012; Barina et al. 2018; Niketić 2021) (Fig. 11). This species was reported from Albania (Barina et al. 2018), but the background of this report has not been examined by us.

##### Notes on taxonomy.

This species with subpatent to patent pubescence was accepted by Cristofolini (1991), but under a wrong name, *C.lasiosemius*, probably because of the unavailability of the type collection of the latter species name.

##### Notes on nomenclature.

Degen (1893) described *Cytisusfrivaldszkyanus* from a few localities in present-day Bulgaria, citing four syntype gatherings. The examined material is fairly homogeneous, and the application of the species name is unambiguous. So far, we refrain from lectotypification because the main collection of Degen at BP has not been examined by us.

*Cytisusrhodopeus* was first mentioned in the synonymy of *C.eriocarpus* by Degen (1893) and validly published by Bornmüller (1925) without any descriptive matter, but with a reference to the description of *C.absinthioides* in Velenovský (1891). Five syntypes from Bulgaria were cited in the original description (Velenovský 1891), which deviated much from the description of the true *C.absinthioides* provided by Janka (1872) by a longer calyx (13–15 mm long vs. 7–8 mm long in *C.absinthioides*) with patent (vs. appressed) hairs.

Through the kindness of P. Mráz, we traced a specimen in the collection of J. Velenovský at PRC, which exactly corresponds to the protologue by its diagnostic characters and taxonomic references on its label (to *C.absinthioides* Janka and “*C.eriocarpus* Boiss. var.”, as Velenovský (1891) also noted a relationship with the latter species). This specimen fully reflects the taxonomic concept of Velenovský (1891) and is designated as a lectotype of *C.rhodopeus* here.

Cristofolini (1991) accepted C.austriacussubsp.microphyllus “(Boiss.) Boiss.” as the correct name for a small-leaved segregate of *C.austriacus*, citing *C.pindicola* (Degen) Halácsy in its synonymy. The type collection of *C.microphyllus* Boiss. is quite dissimilar from *C.pindicola* and belongs to *C.frivaldszkyanus* because of its strong suberect stems, partly obovate (vs. lanceolate) leaflets and pods with nearly patent (vs. appressed) hairs.

### *Cytisuspygmaeus* group

Table 2

**Table 2.** Diagnostic characters in the *Cytisuspygmaeus* group.

	*C.pygmaeus*	*C.eriocarpus*	*C.smyrnaeus*
stems	low (10–20 cm), much branching, hairs 0.3–0.6 (–1.5) mm long, appressed	low (10–20 cm), much branching, hairs 2 mm long, patent	low (10–20 cm), much branching, hairs 0.5–1.0 mm long, appressed to subpatent
leaves	leaflets lanceolate, acute, hairs 0.4–1.0 mm long, appressed	leaflets broadly elliptic to obovate, subrotund, hairs 1.3–1.5 mm long, subpatent	leaflets broadly elliptic to obovate, subrotund, hairs 0.9–1.2 mm long, appressed, sericeous
pedicels	hairs 0.5–0.7 mm long, subpatent	hairs 2–2.5 mm long, patent	hairs 0.5–0.7 mm long, subpatent
calyx	11–14 mm long, hairs 0.5–1.2 mm long, subpatent	10–12 mm long, hairs 2.0–2.5 mm long, subpatent	11–14 mm long, hairs 0.7–1.2 mm long, patent
pods	hairs subappressed	hairs subpatent	hairs subappressed

**Taxonomy.** The diagnostic characters of this species group are mostly prostrate habit and pseudolateral inflorescences. This group is very poorly known and may be an artificial assemblage of superficially similar species. Their distributions need to be verified due to common confusions and misidentifications.

#### 8. Cytisus pygmaeus

Taxon classificationPlantaeFabalesFabaceae

Willd., Sp. Pl., ed. 4, 3(2): 1127 (1802)

6522BE6B-E5FD-5D6C-ACD1-BAD3142C2EB1

**Figure 12.**
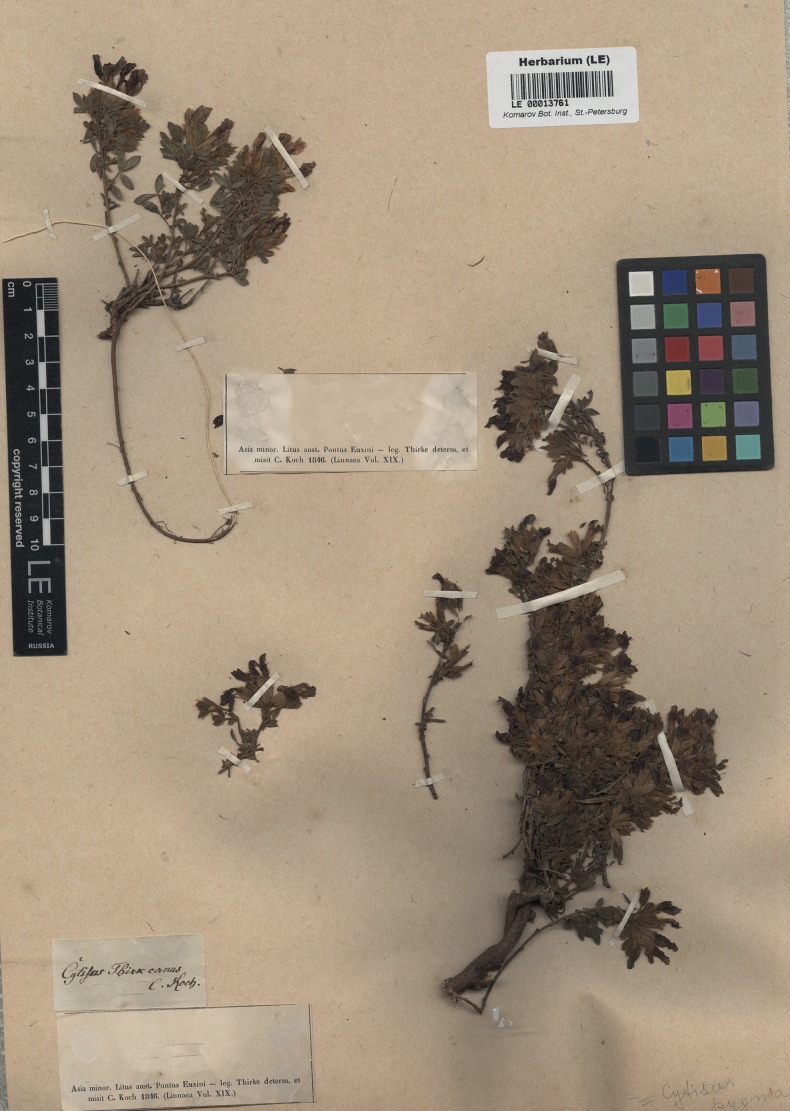
Lectotype of *Cytisusthirkeanus* K.Koch.

– Chamaecytisus pygmaeus (Willd.) Rothm. in Feddes Repert. 53: 144 (1944) – Chamaecytisusaustriacussubsp.pygmaeus (Willd.) Ponert in Feddes Repert. 83: 619 (1973).
= Cytisus tmoleus Boiss., Diagn. Pl. Orient., ser. 1, 2: 11. 1843, syn. nov. – Cytisuseriocarpussubsp.tmoleus (Boiss.) Cristof. in Webbia 45(2): 207 (1991) – Chamaecytisustmoleus (Boiss.) Rothm. in Feddes Repert. Spec. Nov. Regni Veg. 53: 144 (1944). Type. Turkey. “Asia Minor”, *P. Aucher-Éloy 1101* (syntypes K 000829770, P 02952916, 02952919).
= Cytisus chrysotrichus Boiss., Diagn. Pl. Orient., ser. 1, 2: 12 (1843). Type. Turkey. Bursa Province: “In dumosis Olympi Bithyniae” [= Uludağ Mt.], 06.1842, *E. Boissier* (syntypes K 000829766, 000829767, LE 01207296–01207299, NY 1843152).
= Cytisus thirkeanus K.Koch in Linnaea 19(1): 61 (1846). Type. Turkey. Trabzon Province: “Asia minor. Litus australis Pontus Euxini”, [1843], *Thirke* (lectotype LE 00013761, designated here; isolectotypes LE 00013760, G-Boiss 00365031). Fig. 12.

##### Type.

Turkey. [Galatia], *D. Sestini* (lectotype B-Willd 13632-010, designated by Pifkó and Barina (2016: 172); isolectotype HAL 0100154).

##### Distribution.

**Figure 13.**
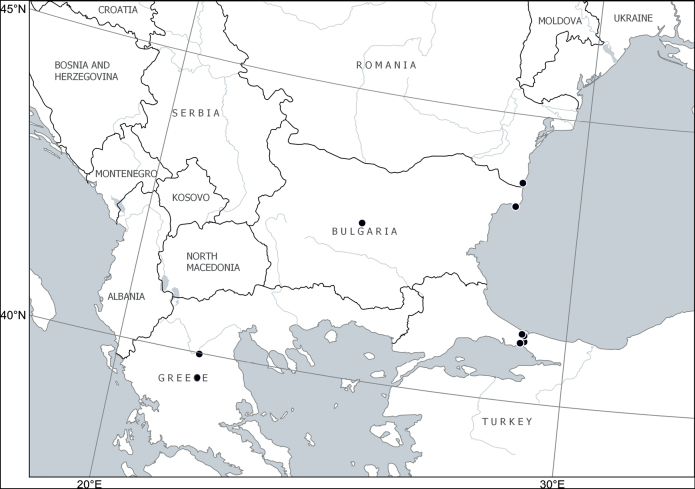
Distribution of *Cytisuspygmaeus* Willd.

European and Asiatic Turkey, Bulgaria, Greece (Kuzmanov 1976; Cristofolini 1991; Assyov and Petrova 2012), Romania (Fig. 13). Other European records, from North Macedonia and Serbia (Diklić 1972; Micevski 2001), seem to belong mostly to *C.jankae* or *C.calcareus*. A record of *C.jankae* from Romania (Grinţescu 1957) is treated as belonging to *C.pygmaeus* here.

##### Notes on taxonomy.

The leaves of this species may vary slightly from oblong-lanceolate to oblanceolate. Plants with the leaves looking more lanceolate were described as *C.pygmaeus* and *C.chrysotrichus*, whereas plants with rather oblanceolate leaves were named *C.tmoleus* and *C.thirkeanus*. This difference, albeit very subtle, led Cristofolini (1991) to classify *C.pygmaeus* as a subspecies of *C.austriacus*, whereas he placed the plants described as *C.tmoleus* to *C.eriocarpus*. Having examined some material from Asiatic Turkey, we observed both types of leaves in the same plants; this makes the distinction practically impossible.

The pubescence on calyces of *C.pygmaeus* is variable, ranging from semi-patent to subappressed. The type collection of *C.pygmaeus* has clearly semi-patent hairs.

Niketić (2021) provisionally accepted the occurrence of *C.pygmaeus* in Serbia, although the relevant materials have not been examined. Micevski (2001) listed it among doubtful records in North Macedonia. The collections identified as *C.pygmaeus* which we examined from the Balkans belong to *C.jankae*, and we assume that the distribution of *C.pygmaeus* in Europe may be much more limited than it is currently believed.

##### Notes on nomenclature.

Willdenow (1802) described the species without mentioning floral characters. His indication of “Galatia” in the protologue corresponds to the fruiting specimen of D. Sestini in Willdenow’s personal collection. A duplicate of this collection was separated to HAL, which allowed Pifkó and Barina (2016) to designate a lectotype at B.

The synonymy above was established already by Boissier (1872), except for the placement of *C.tmoleus*, which he considered to differ in a denser, sericeous indumentum of the plant. According to our observations, the density of indumentum in *C.pygmaeus* may look variable, depending on ecological conditions, and the plants described as *C.tmoleus* can be regarded as an extreme variant.

#### 9. Cytisus eriocarpus

Taxon classificationPlantaeFabalesFabaceae

Boiss., Diagn. Pl. Orient., ser. 1, 2: 11 (1843)

7D41E3CB-D718-58E7-ADEA-C2EEEE344762

– Cytisus supinus subsp. eriocarpus (Boiss.) Stoj. & Stef., Fl. Bulg. 2: 624 (1925) – Chamaecytisuseriocarpus (Boiss.) Rothm. in Feddes Repert. 53: 144 (1944).

##### Type.

Turkey. İzmir Province: “Tmolus ad Bozdagh”, 06.1842, *E. Boissier* (K 000829776, lectotype designated by Gibbs (1970: 17); isolectotypes BM 000630427, E 00296045, GOET 005097, K 000829774, KW, LE 01207308, 01207311, 01207312, MEL 2347576, NY 01843146, P 02952858).

##### Distribution.

Asiatic Turkey. European records (Cristofolini 1991; Barina et al. 2018) may be erroneous due to the synonymisation or inclusion of *C.absinthioides* and *C.frivaldszkyanus*.

##### Notes on taxonomy.

This species is very similar to *C.frivaldszkyanus* due to its abundant patent pubescence. However, it differs from the latter in its broadly elliptic to obovate, nearly rotund leaflets, which are apically subrotund (vs. elliptic-lanceolate to obovate, broadly acute in *C.frivaldszkyanus*). *Cytisuseriocarpus* is similar to *C.hirsutus*, from which it differs in its pubescence (abundant short hairs mixed with long patent hairs vs. only long patent hairs in *C.hirsutus*) and smaller subrotund leaflets, as already noted in the protologue (Boissier 1843).

##### Notes on nomenclature.

Gibbs (1970) inadvertently designated a specimen at K as the lectotype of *C.eriocarpus*.

#### 10. Cytisus smyrnaeus

Taxon classificationPlantaeFabalesFabaceae

Boiss., Diagn. Pl. Orient., ser. 1, 2: 10 (1843)

0CA99F06-300F-5F0D-8C74-955F973CEF6C

##### Type.

Turkey. “Montes Smyrnae”, 06.1842, *E. Boissier* (syntypes BP 208133, E 00296047, FR 003144, GOET 005096, JE 00014575, 00014576, 00014577, K 000829774, KW, MEL 2347575, P 02952937, 02952942, 02952944, 02952950, 02952951, 02952952, JE 00014575, 00014576, 00014577, W 9918, 0031010).

##### Distribution.

Asiatic Turkey.

##### Notes on taxonomy.

*Cytisussmyrnaeus* is a poorly known species, probably endemic to Asiatic Turkey. It is most closely similar to *C.eriocarpus*, from which it differs by the lack of patent hairs on its stems and pedicels (Pifkó and Barina 2016).

Gibbs (1970) and Cristofolini (1991) added *C.smyrnaeus* to the synonymy of *C.eriocarpus*, which was treated broadly and included plants with different kinds of pubescence.

## Supplementary Material

XML Treatment for Cytisus hirsutus

XML Treatment for Cytisus hirsutus var. ciliatus

XML Treatment for Cytisus polytrichus

XML Treatment for Cytisus polytrichus var. subglabratus

XML Treatment for Cytisus austriacus

XML Treatment for Cytisus jankae

XML Treatment for Cytisus calcareus

XML Treatment for Cytisus absinthioides

XML Treatment for Cytisus frivaldszkyanus

XML Treatment for Cytisus pygmaeus

XML Treatment for Cytisus eriocarpus

XML Treatment for Cytisus smyrnaeus

## Additional information

### Author ORCIDs

Alexander N. Sennikov https://orcid.org/0000-0001-6664-7657

Valery N. Tikhomirov https://orcid.org/0000-0003-1822-0557

### Data availability

The dataset of distributional records collected for the present work was published through the Internet Archive (available online: https://archive.org/details/cytisus-balkans).
